# Self-microemulsifying drug delivery system (SMEDDS) of curcumin attenuates depression in olfactory bulbectomized rats

**DOI:** 10.1016/j.heliyon.2020.e04482

**Published:** 2020-08-10

**Authors:** Manoj Aswar, Mangesh Bhalekar, Akshata Trimukhe, Urmila Aswar

**Affiliations:** a Department of Pharmacology, Sinhgad Institute of Pharmacy, Narhe, Pune, Maharashtra, India; b Department of Pharmaceutics, AISSMS College of Pharmacy, Pune, Maharashtra, India; c Department of Pharmacology, BVDU, Poona College of Pharmacy, Pune, Maharashtra, India

**Keywords:** Pharmaceutical science, Pharmacology, Toxicology, Depression, Curcumin, Olfactory bulbectomy, SMEDDS

## Abstract

**Background:**

Current therapies for depression remain limited and plagued by various side effects. Problems associated with curcumin administration include poor aqueous solubility and bioavailability issues. Hence to overcome these, curcumin self micro emulsifying drug delivery system (SMEDDS) which will result in a nanosize emulsion droplets when administered *in vivo* were formulated in the present study.

**Methods:**

Depression was induced by bilateral olfactory bulbectomy and the animals were randomized into 8 groups as normal, control [(vehicle 10 ml/kg, p.o., (per oral)], pure curcumin (10, 20, 40 mg/kg, p.o.), and curcumin SMEDDS (10, 20, 40 mg/kg, p.o). After 14 days of respective treatment, behavioral parameters such as open field test (OFT), ambulation counts and passive avoidance response (PAR) were evaluated. At the end of experiments, blood was withdrawn from r.o.p (retro orbital plexus) for serum cortisol estimation.

**Results:**

In OFT, increased central area frequency, peripheral area frequency, central area duration and decreased rearing and grooming were recorded with an increased ambulation counts. In PAR, significant reduction in number of trials and step down from platform was observed in the animals treated with test drug. Serum cortisol level was also found to be decreased in the test groups.

**Conclusion:**

Behavioral and biochemical estimations in the present study revealed the improved brain permeability and further increase in biological activity of curcumin SMEDDS.

## 1 Introduction

The term psychiatric illness (or mental illness) encompasses a broad range of medical conditions affecting thinking, feeling, mood, ability to relate to others and daily functioning within society. Such conditions include schizophrenia, psychosis, depression, bipolar affective disorder, anxiety disorders (e.g. panic disorder, obsessive-compulsive disorder and post-traumatic stress disorder) and disorders relating to substance abuse [1]. As per WHO, major depression represents the most common mental health problem worldwide with an estimated 322 million people, equivalent to 4.4% of the world's population [2]. Most common clinical manifestation includes feeling of intense sadness, worthlessness or excessive guilt. Symptomatically, a significant increase or decrease in appetite, loss of interest or pleasure and in worsening conditions, suicidal ideation or suicidal attempts [3]. The clinical manifestation of different types of depression may be diverse but all these types of depression ultimately affect mood or thoughts [4, 5]. Selective Serotonin Reuptake Inhibitors (SSRIs') are the mainstay for the management of depression [6, 7]. But existing drug therapies are linked with adverse effects like anorexia, decreased libido, activation and aggravation of psychosis, serotonin syndrome, cheese reaction etc. [8]. Hence, plant derived products are increasingly being sought out as an option to avert the adverse effects with an existing therapy. St John's Wort (*Hypericum perforatum L.*) is a drug from natural origin which is now accepted as the classified antidepressant drug [9, 10].

Turmeric is most widely used as flavoring and coloring agent in various Indian dishes. It has a wide biological and pharmacological profile as it is reported to possess anti-oxidant, anti-inflammatory and anti-carcinogenic properties [11, 12]. It also possess hypocholesterolemic, antibacterial, wound healing, antispasmodic, anticoagulant, antitumor and hepatoprotective activities [13]. Curcumin from *Curcuma longa L.* has also been reported for its potent antidepressant activity [14, 15, 16]. Curcumin is an inhibitor of monoamine oxidase (MAO) enzyme and also modulates the levels of norepinephrine, dopamine, and serotonin in the brain [15, 16]. Curcumin being poorly water soluble for about 0.011 mg/ml, when administered orally, major portion is excreted through faeces and only small portion is absorbed within the intestine [17]. The absorbed curcumin undergoes rapid metabolism in the liver and plasma and is extensively converted to its water soluble metabolites (glucuronides and sulphates) and excreted through urine [18] which results in poor bioavailability [18]. The ability of curcumin and its nano-formulation to cross the Blood Brain Barrier (BBB) are closely correlated with its hydrophobic property. Nanoparticle formulation has significantly increased the retention time of curcumin in the cerebral cortex (increased by 96%) and hippocampus (increased by 83%) as compared to pure curcumin [19]. Hence, self-micro emulsifying drug delivery system (SMEDDS) of curcumin nanoparticles was formulated to overcome the problems associated with oral absorption and poor bioavailability.

Olfactory bulbs transmit the sense of smell to the frontal cortex of the brain by transduction pathway where the physical stimulus (smell) is converted into action potential. Bilateral removal of the olfactory bulbs (OBX) elicits a variety of behavioral, neurochemical, neuroendocrine, and neuroimmune alterations, many of which mimic the symptoms of depression [20]. Several authors have used this model in rats primarily for the detection of antidepressive properties [16, 21, 22, 23]. Even before its suggestion as a model for depression, the OBX model has been studied for changes in sexual behavior [24], food intake and preference [25], as well as effects of handling [26], maternal behavior [27] and nursing behavior [28]. Consequently in the present study, bilateral olfactory bulbectomy model was used for the induction of depression in rats.

## 2 Materials and methods

### 2.1 Preformulation studies

Curcumin was dissolved in ethanol to produce (100 μg/ml) stock solution which was suitably diluted to produce final concentrations of 2, 4, 6, 8, 10 μg/ml. The UV absorbance was noted at λ max of 400 nm to plot the calibration curve.

### 2.2 Determination of solubility of curcumin

The solubility of curcumin in oil, surfactant and co-surfactant was determined by adding an excess amount of curcumin to 5 ml of excipient separately. The mixtures were then shaken for 48 h at 25 ± 0.5 °C in an orbital shaker. The equilibrated samples were centrifuged at 3500 rpm for 15 min. The supernatant was diluted with ethanol analyzed for curcumin content using validated UV- visible spectrophotometer. All measurements were done in triplicate.

### 2.3 Pseudoternary phase diagram

Pseudoternary phase diagram was prepared by titrating oleic acid as oil phase with tween 80 and propylene glycol as surfactant/co-surfactant at ratio 1:1, 1:2 and 2:1 (v/v), The pseudoternary phase diagram were constructed by titrating above mixture with double distilled water and visually observed for phase clarity. Nine different combinations of oil and Smix, 1:9, 2:8, 3:7, 4:6, 5:5, 6:4, 7:3, 8:2, 9:1 were made so that maximum ratios were covered for the study to demarcate the boundaries of phases precisely formed in the phase diagrams. Pseudo-ternary plots were constructed using CHEMIX school trial version software 3.5 [29].

### 2.4 Formulation of curcumin SMEDDS

Comparison of phase diagrams with emulsion forming maximum area and uniform size distribution of the globules was selected. Accurately weighed curcumin was placed in a glass vial and oleic acid, tween 80 and propylene glycol in desired ratio were added to the vial and was sonicated for 15 min for solubilization of drug. The formulation was stored at room temperature until further use.

### 2.5 Characterization of curumin SMEDDS

#### 2.5.1 Zeta potential

The zeta potential of 1% dispersion of formulation in water was measured using Malvern Zetasizer ZS 90 UK.

#### 2.5.2 Particle size analysis

The particle size analysis was performed on Nanophox (Sympatec, Germany).

#### 2.5.3 Ex-vivo study

Rat intestinal membrane was used to determine the drug release study. Intestine (length postion s/a proximal) was washed with saline to remove excretory product present in the intestine by flushing. Two intestines of equal size around 8 cm were taken and one was filled with curcumin SMEDDS and the other with pure curcumin in phosphate buffer (pH 6.8). These two intestinal segments were separately tied using nylon thread and assembled into two different organ bath with aeration. The release study was performed in 50 ml of phosphate buffer at 37 ± 0.5 °C. 2 ml sample was withdrawn at regular intervals i.e. 10, 20, 30, 40, 50 and 60 min and aliquot amount of phosphate buffer was replaced in order to maintain sink condition. The withdrawn samples were analyzed for drug content using UV-visible spectrophotometer at 400 nm [30].

### 2.6 Experimental animals

Adult Wistar rats of either sex (250–350 g) were housed at Institute Animal House in groups of six animals per cage at standard laboratory condition with a temperature of 25 ±1°c, relative humidity of 45–55%. All rats were allowed to access to food and water *ad libitum*. The 12hrs light/dark cycle was maintained.

### 2.7 Approval of experimental protocol

The experimental protocol was approved by Institutional Animal Ethical Committee (IAEC) of Sinhgad institute of pharmacy, Narhe, Pune, constituted as per the committee for purpose of supervision and control on the experimental animal CPCSEA reg. no 1139/PO/a/07/CPCSEA. The approved protocol number is SIOP/IAEC/2017/02/06.

### 2.8 Induction of depression by olfactory bulbectomy

For bilateral olfactory bulbectomy, rats were anaesthetized with ketamine (80 mg/kg) and Xylazine (5 mg/kg) through intra-peritoneal route. The animal was placed in streotaxic frame, head was shaven and midline scalp sagittal incision (1 cm) was made. Bilateral burr holes were drilled (2 mm diameter), 8 mm anterior to bregma and 2 mm lateral from midline. The olfactory bulbs from both the burr were aspirated using a blunt hypodermic needle without damaging frontal cortex. The burr holes were then filled with haemostatic sponge to prevent bleeding. The incision was sutured and topical soframycin gel was applied to that region. Intramuscularly, diclofenac sodium (2 mg/kg) was administered as analgesic [22]. The animals were housed in an individual cage and observed for 15 days and further on 16^th^ day, the animals were grouped as per the protocol.

### 2.9 Experimental design

The animals were randomly divided into eight groups consisting of six animals in each group. The animals received drug treatment once a day through oral administration for 14 consecutive days. The control group received vehicle (10 ml/kg). The standard group received pure curcumin (PC, 10, 20, 40 mg/kg) while the test group received curcumin SMEDDS (CS, 10, 20, 40 mg/kg).

### 2.10 Behavioral parameters

After 14 days of treatment with respective drugs various behavioral parameters such as passive avoidance test, open field test and ambulation counts were evaluated.

#### 2.10.1 Passive avoidance test

The passive avoidance apparatus consists of open box (50 × 50 × 50 cm^3^) with stainless steel grid floor. The electrified rods were connected to the terminals of a shock generator that delivered a constant voltage. The intensity of delivered shock was 0.75 mA which lasted for 1 s. Rats were subjected to step-down passive avoidance training and testing on the 14th day of chronic curcumin administration. A platform (12 × 12 × 4 cm^3^) can be inserted through one side wall to allow a jump-up escape response. Each rat was placed on this platform and when it stepped off the platform with all four paws it received an electric shock. The animal was immediately removed from the experimental apparatus and placed in its home cage. After 30 s, the next trial was initiated. Each rat was trained until it learned to remain on the platform for 1 min [16].

#### 2.10.2 Open field test

Open field test apparatus consists of a square arena (60 × 60 cm) with white floor divided into 36 squares (10 × 10 cm). In this test, the 20 squares adjacent to the wall represented the protected field named ‘arena periphery’ while the other 16 squares represented an exposed field named ‘arena centre’. The test was initiated by placing a single rat in the middle of the arena and letting it move freely for 5 min and the behavior continuously video-graphed (V J instruments, India) [31]. The following parameters were evaluated during this 5 min session:

a) Central area frequency
b) Peripheral area frequency
c) Central area duration
d) Rearing
e) Grooming

#### 2.10.3 Ambulation counts by actophotometer

Locomotor activity (ambulations) was determined by using actophotometer. The apparatus is equipped with 6 photocells at the bottom. Each animal was observed for a period of 5 min in a square closed field (30 × 30 × 30 cm). When the rat breaks the beam of light, it activates the digital counter where locomotion was expressed in terms of total number of ambulations [14].

### 2.11 Serum cortisol estimation

At the end of experiments, blood sample was collected through r.o.p for serum cortisol estimation. Rats were sacrificed on 16^th^ day, brains were excised on 16^th^ day with high dose of anesthesia and brains were excised quickly and stored at -25 °C. Serum cortisol was assayed by chemi luminescent immuno assay (C.L.I.A) using Cortisol kit (Cusabio, USA, Catalog No. CSB-E05112r).

### 2.12 Statistical analysis

Data for each parameter was analyzed by one-way ANOVA followed by Dunnet's post hoc test using a graph pad, prism software, version 5.0, USA.

## 3 Results

### 3.1 Preformulation studies

#### 3.1.1 Calibration curve of curcumin in ethanol

**Figure 1:**
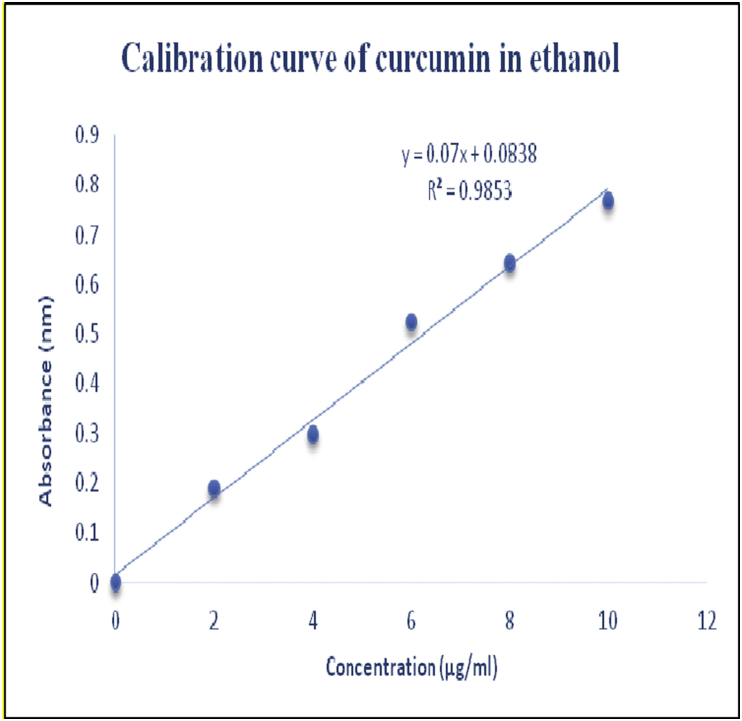
Calibration curve of curcumin in ethanol.

The curcumin obeyed Beers law in the concentration range 2–10 μg/ml with high correlation coefficient R^2^ = 0.9853. The regression equation was found to be y = 0.07x + 0.0838 (Figure 1).

#### 3.1.2 Solubility of curcumin

**Figure 2:**
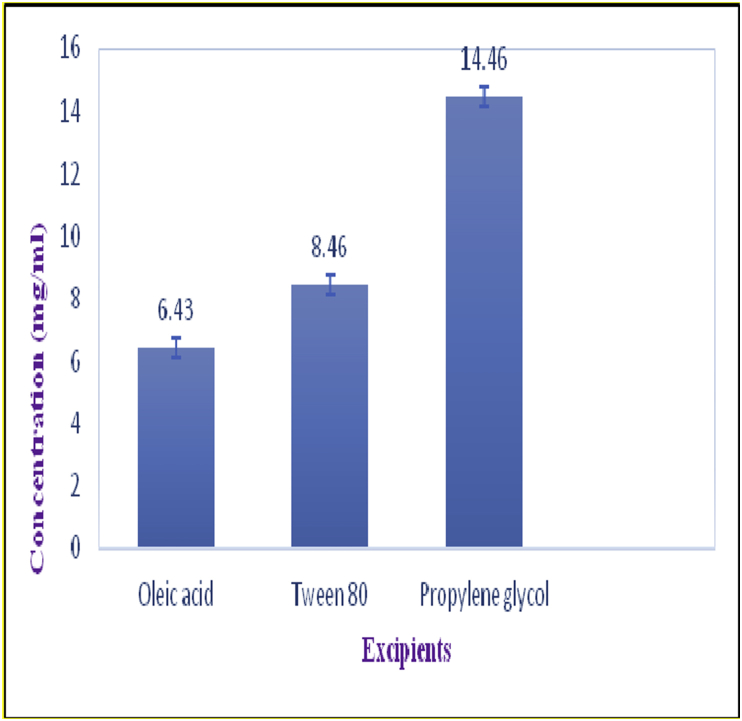
Solubility of curcumin in excipient.

The solubility of curcumin in oleic acid (oil) was found to be 6.43 mg/ml whereas in tween 80 (surfactant) and propylene glycol (co-surfactant) it was found to be 8.46 mg/ml and 14.46 mg/ml respectively (Figure 2).

### 3.2 Pseudoternary phase diagram of SMEDDS

**Figure 3:**
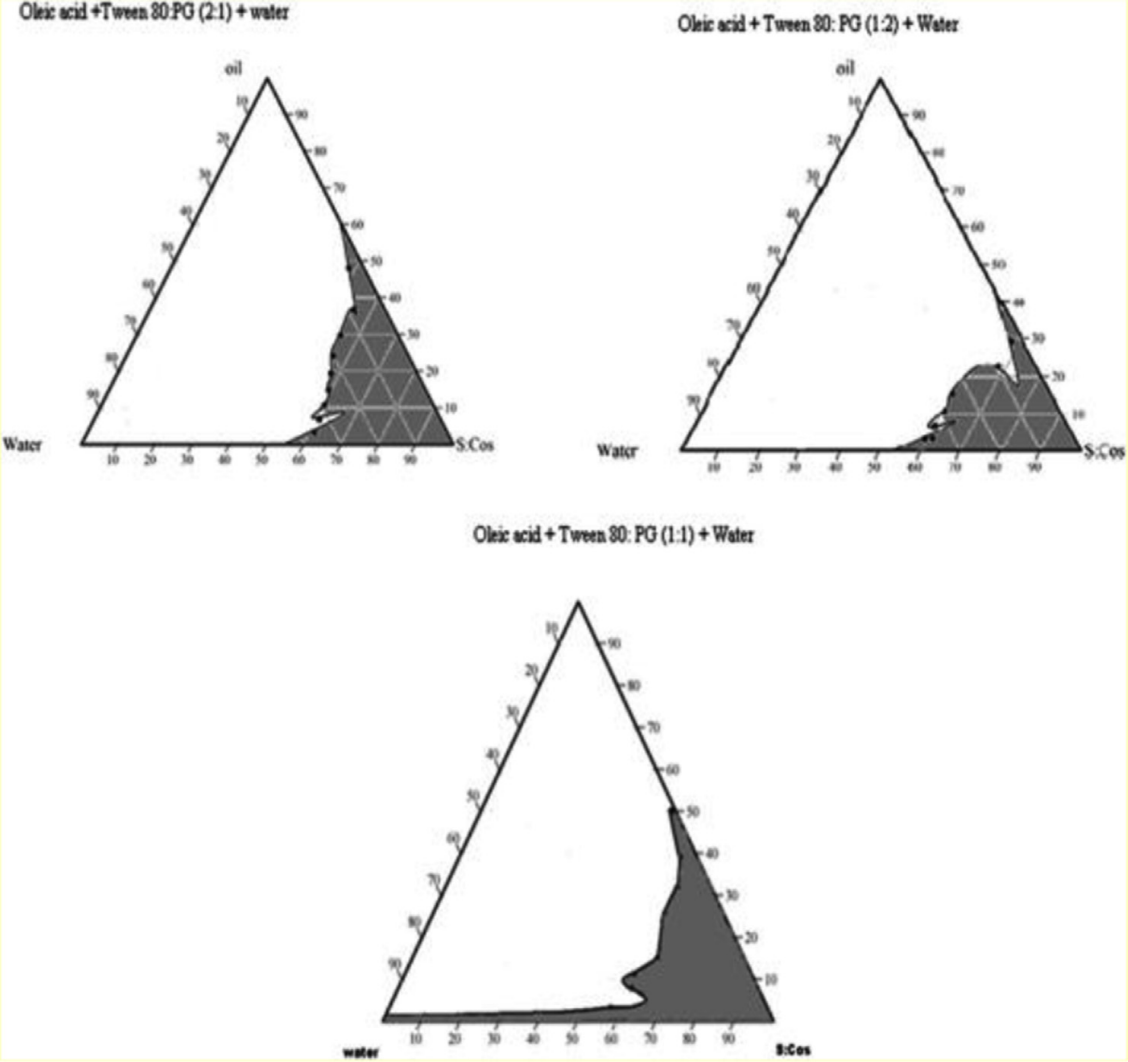
Pseudoternary phase diagrams.

The titration values obtained from the three ratios of oleic acid + Smix (tween 80: propylene glycol) i.e. 1:1, 1:2 and 2:1 were analyzed in the CHEMIX school trial version software 3.5 for maximum area. The maximum area was obtained in the ratio 1:1 as compared to the other ratios (Figure 3).

### 3.3 Characterization of curcumin SMEDDS [32]

#### 3.3.1 Zeta potential

**Figure 4:**
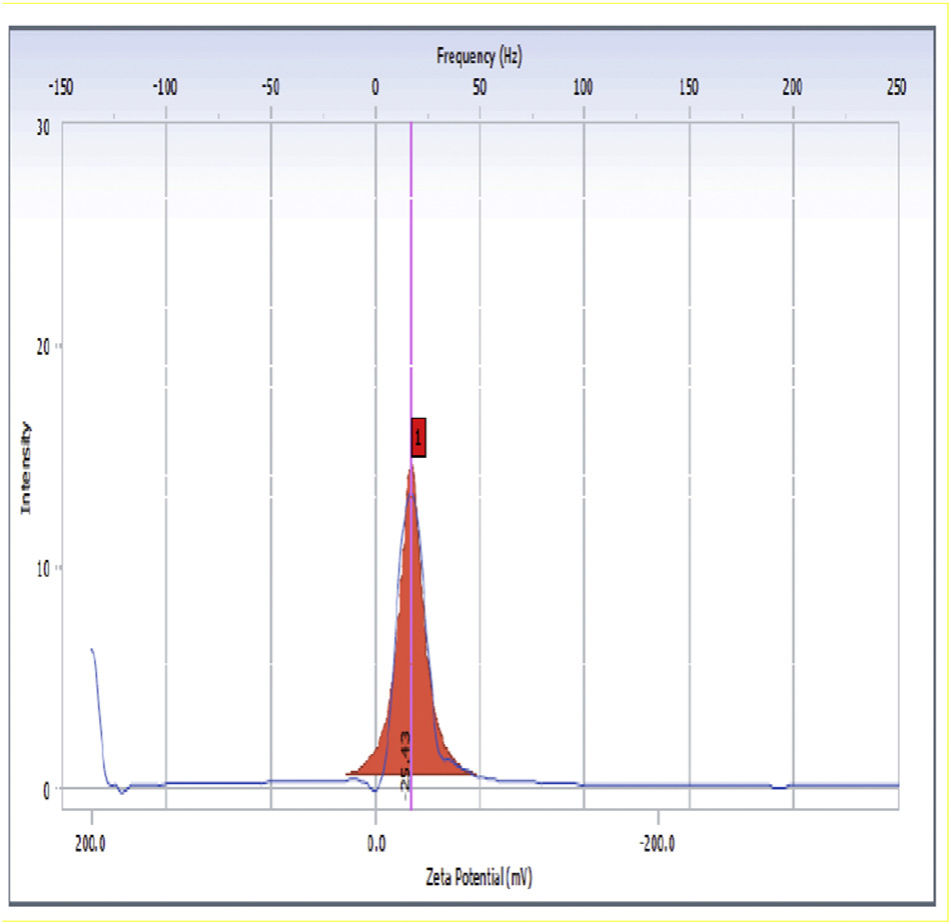
Zeta potential of curcumin SMEDDS.

Using oleic acid as oil and tween 80 as surfactant with co-surfactant propylene glycol, the emulsion obtained was negatively charged with zeta potential – 25.43 ± 0.937 mV (Figure 4).

#### 3.3.2 Particle size analysis

**Figure 5:**
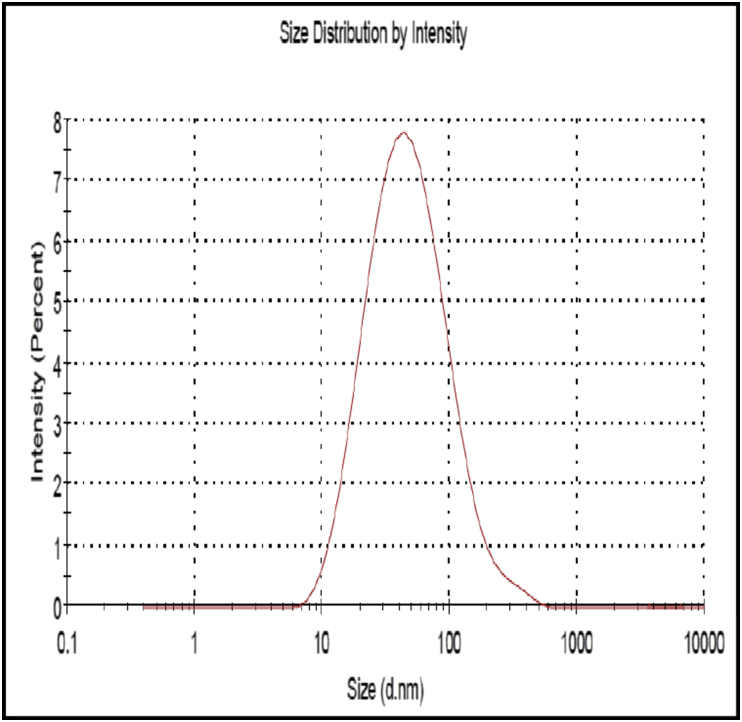
Particle size analysis of curcumin SMEDDS.

The globule size obtained was 44.13 ± 0.695 nm. The polydispersity index (PDI) was found to be 0.446 (Figure 5).

#### 3.3.3 Ex-vivo study

**Figure 6:**
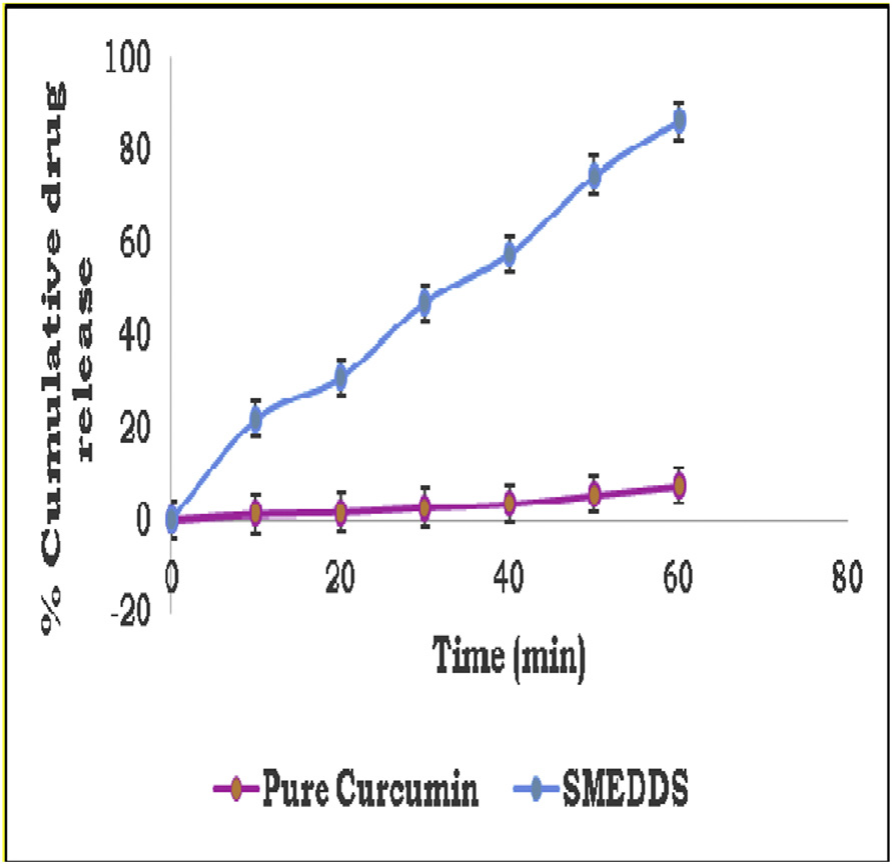
Percent cumulative drug release graph.

The samples of pure curcumin and curcumin SMEDDS withdrawn at regular intervals i.e. 10, 20, 30, 40, 50 and 60 min were analysed for drug content using UV-visible spectrophotometer at 400 nm. The drug release of pure curcumin was found to be less as compared to the curcumin SMEDDS after 10 min and was observed maximum at 60 m (Figure 6).

### 3.4 Behavioral parameters

#### 3.4.1 Passive avoidance

**Figure 7:**
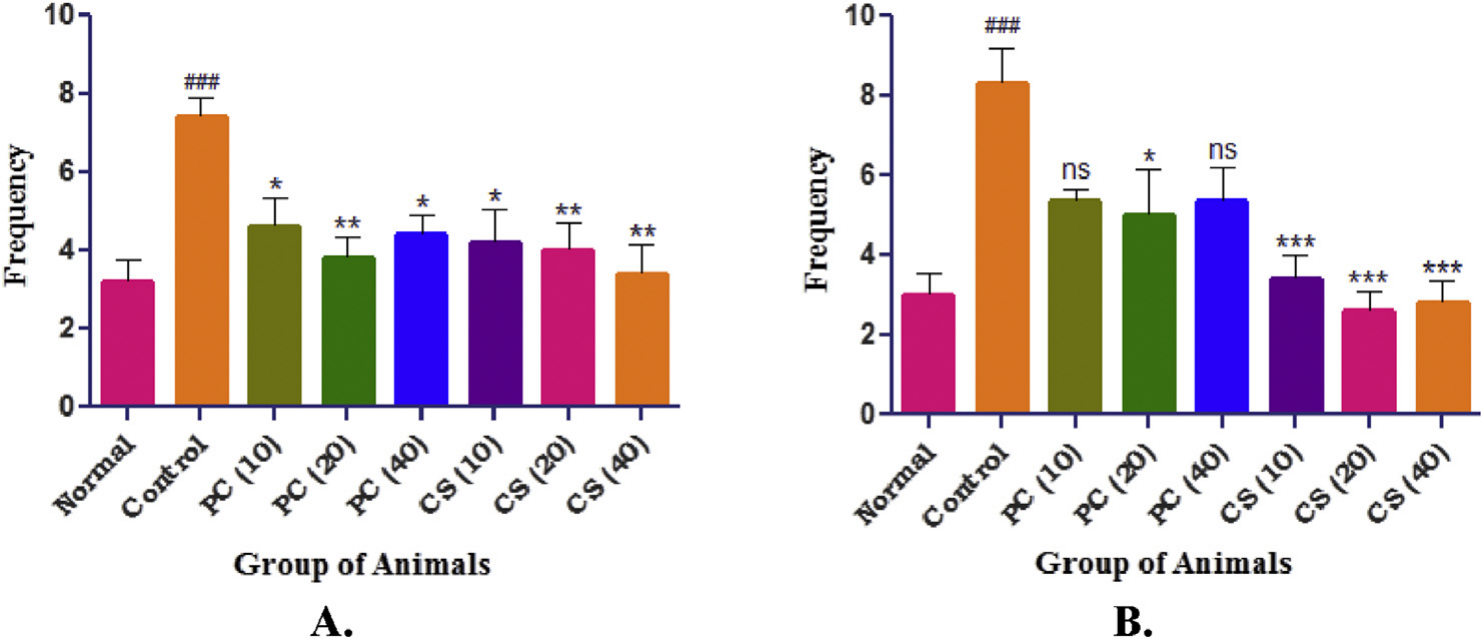
Effect of PC and CS passive avoidance response. A. No. of Trials, B. Step down from platform. Data was expressed as mean ± SEM (n = 6) and was analyzed by one way ANOVA followed by Dunnett's test. Values in parenthesis indicates dose in mg/kg, ^###^p < 0.001 as compared with normal, ∗∗∗p < 0.001 and ∗p < 0.05 as compared with control, ns: non-significant.

A significant increase in number of trials was observed in control group, which was reduced in pure curcumin (10, 20, 40 mg/kg) groups and curcumin SMEDDS (10, 20, 40 mg/kg) groups when compared to control group. Similarly step down from platform in control group was significantly increased as compared to normal group. Non-significant reduction was observed in pure curcumin (10 and 40 mg/kg) groups while significant reduction was observed in curcumin SMEDDS (10, 20, 40 mg/kg) groups as compared to control group (Figure 7).

#### 3.4.2 Open field test

**Figure 8:**
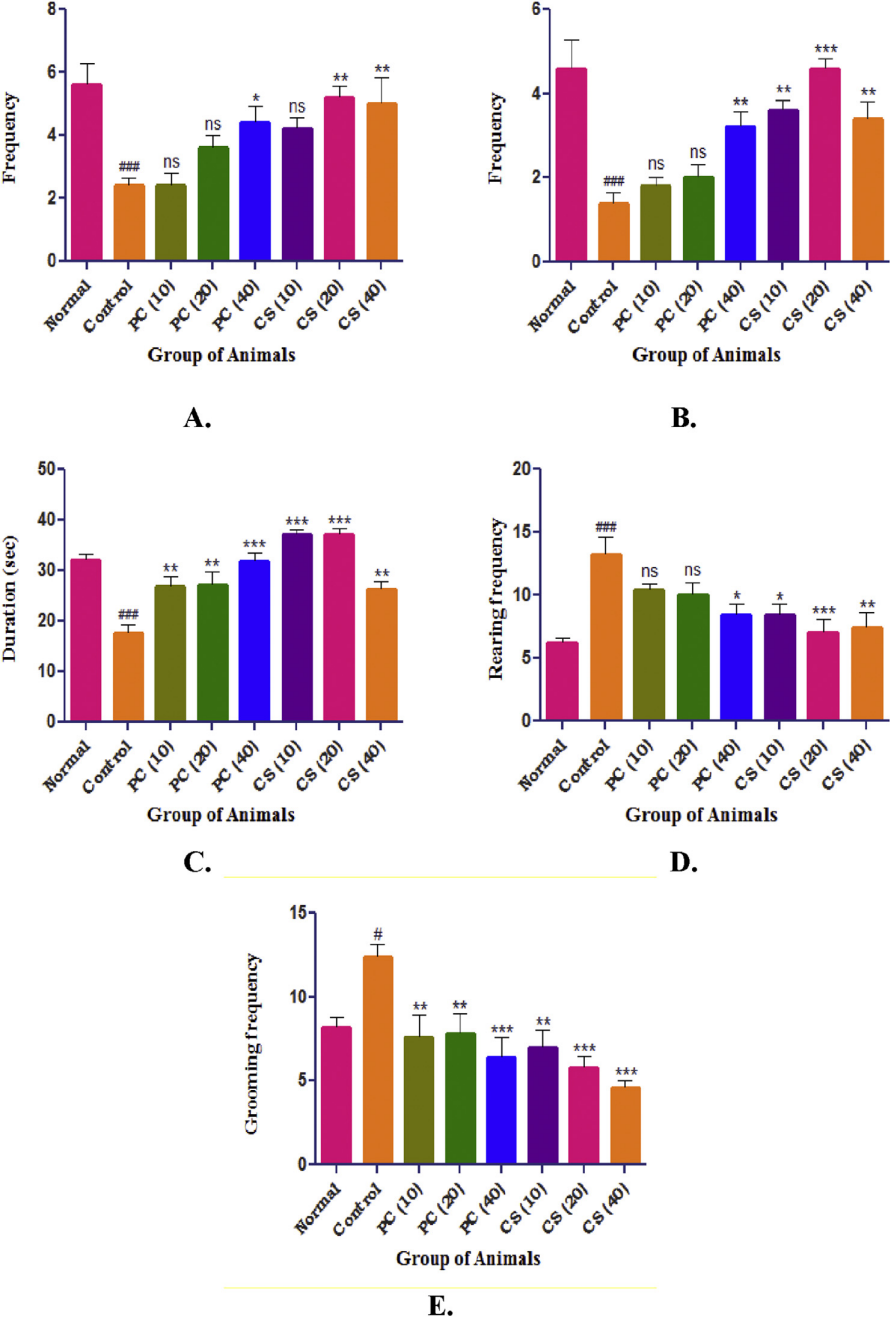
Effect of PC and CS on locomotion in open field test. A. Central Area Frequency, B. Peripheral area frequency, C. Central area duration, D. Rearing, E. Grooming. Data was expressed as mean ± SEM (n = 6) and was analyzed by one way ANOVA followed by Dunnett's test. Values in parenthesis indicates dose in mg/kg, ^#^p < 0.05 as compared with normal and ∗∗p < 0.01, ∗∗∗p < 0.001 as compared with control.

Movement of animals in the central area frequency and the time spent in the central area was reduced in control group as compared to normal group. Treatment with pure curcumin (10 and 20 mg/kg) non-significantly increased the same; whereas significant increase in frequency and duration was noted in curcumin SMEDDS (20 and 40 mg/kg) groups. Similarly significant reduction in peripheral area frequency was observed in control group as compared with normal group which was again non-significantly increased in pure curcumin (10, 20 mg/kg) groups and significantly increased in curcumin SMEDDS (10, 20, 40 mg/kg) groups. Rearing and grooming was significantly increased in control group as compared to normal group which was further non-significantly reduced in pure curcumin (10, 20 mg/kg) and significantly reduced in curcumin SMEDDS (10, 20, 40 mg/kg) groups (Figure 8).

#### 3.4.3 Ambulation counts by actophotometer

**Figure 9:**
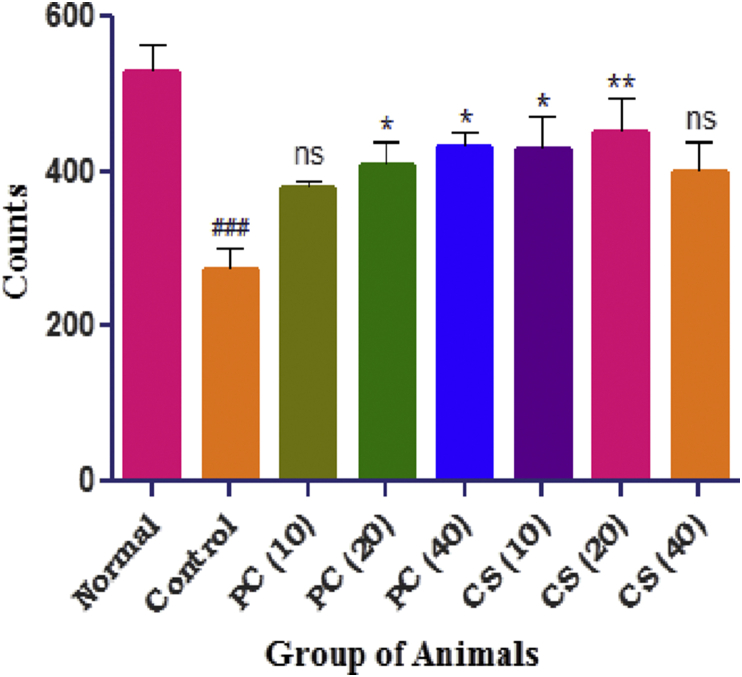
Effect of PC and CS on ambulation counts. Data was expressed as mean ± SEM (n = 6) and was analyzed by one way ANOVA followed by Dunnett's test. Values in parenthesis indicates dose in mg/kg, ^###^p < 0.001 as compared with normal, ∗p < 0.05, ∗∗p < 0.01 as compared with control.

The movement of animals in the control group was significantly reduced whereas treatment either with pure curcumin (20, 40 mg/kg) and curcumin SMEDDS (10, 20 mg/kg) groups significantly increased the count when compared with control group (Figure 9).

### 3.5 Estimation of serum cortisol

**Figure 10:**
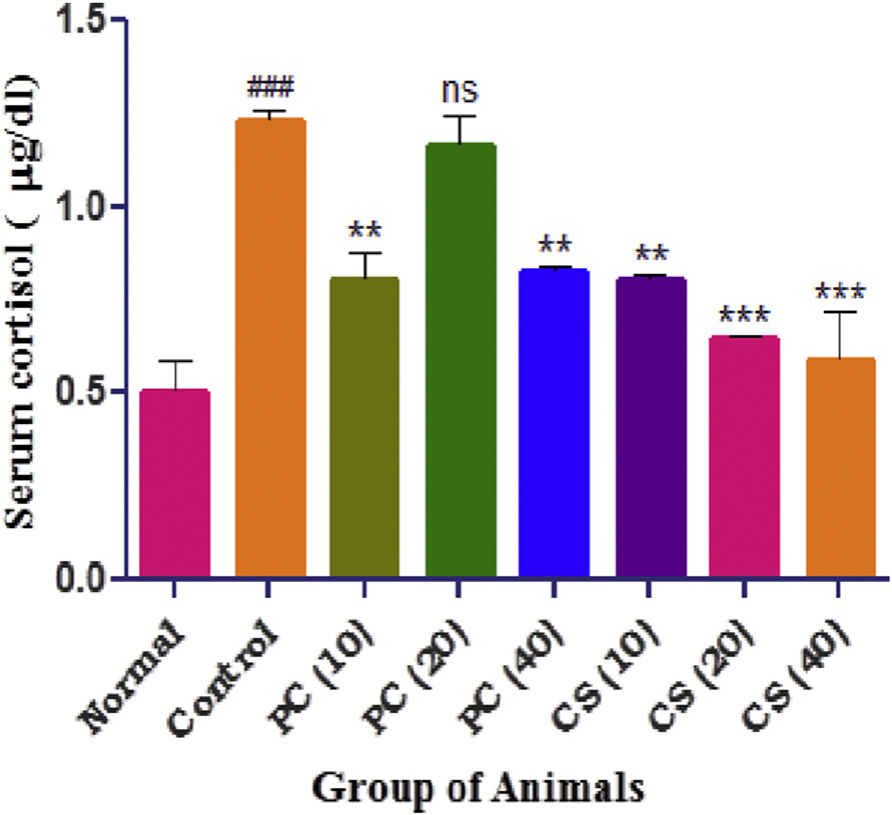
Effect of PC and CS on serum cortisol level. Data was expressed as mean ± SEM (n = 3) and was analyzed by one way ANOVA followed by Dunnett's test. Values in parenthesis indicates dose in mg/kg, ^###^p < 0.001 as compared with normal, ∗∗p < 0.01, ∗∗∗p < 0.001 as compared with control.

Serum cortisol was significantly increased in the animals of control group as compared to normal group. Significant reduction in cortisol level was observed in both the treatment groups (Figure 10).

## 4 Discussion

Pharmacological management of depression is a major challenge because the cause and pathology of depression are still unknown. The rate and extent of oral drug absorption of available drugs for depression are often limited by their poor dissolution and solubilization within the gastrointestinal tract [33]. Multiple studies have reported that, even with high doses of curcumin, the levels of curcumin as well as its *in vivo* metabolites are extremely low in serum and tissues after a short period of time [34, 35, 36]. The application of nanoformulation approaches to currently available drugs is a viable option for optimizing oral drug delivery and maximizing treatment efficacy. SMEDDS are homogenous and isotropic mixture of drug, oil, surfactant and co-surfactant with particle size 10–100 nm. They are used to overcome problems such as low aqueous solubility, low permeability, high molecular weight, pre-systemic first pass-effect, enzymatic degradation, gastric irritation, enhanced bioavailability and stability of drugs [33]. Curcumin being a poor water soluble drug, at about 0.011 mg/ml it shows poor oral absorption and decreased oral bioavailability [37]. Hence, the problems associated with oral administration of pure curcumin can be overcome with formulation of nanoparticles in the form of SMEDDS. These SMEDDS also increases the brain permeability and improved pharmacological activity [18, 19]. The small particle size, presence of surfactants and the anionic zeta potential must have increased the absorption of curcumin and may have also contributed to improved transport across blood brain barrier. For the induction of depression in rats, there are various animal models such as tail suspension test, forced swim test, olfactory bulbectomy and stress induced model. But the bilateral olfactory bulbectomy has garnered attention as a most efficient animal model of depression [20, 38, 39], as this model is based on the hypothesis that removal of the olfactory bulbs affects extensive efferent neuronal networks and disturbs the connection and function of the whole limbic system [38]. The limbic circuit is essential for the maintenance of mood, emotional and memory components of behaviour and hence OBX model was selected in the present study.

Passive avoidance test, Open field test and Ambulation counts plays pivotal role to study memory, cognition and locomotion in animals. In passive avoidance test, training was given to the animals to remain on the platform which indicates the determination of memory and cognition in animals. In the present study, it was observed that the control group required more number of trials as compared to the standard (pure curcumin) and test (curcumin SMEDDS) groups acquiring less number of trials which indicates increased memory and cognition which are in accordance with the previous findings [15, 16].

In open field test, parameters such as central area frequency, peripheral area frequency, central area duration, rearing and grooming were evaluated. Animals in control group showed decreased total number of entries and also the duration of time spent in central area, this represents fear and anxiety like behavior in animals which are the mainstay symptoms of depression. High cortisol levels as observed in current study has been accounted for generalized anxiety. In the standard and test groups, the number of entries and time spent in central area was significantly increased and it was more significant in test group indicating anti-anxiety effects by curcumin SMEDDS which are in agreement with the previous reports [15, 16, 31].

Ambulation count in the animals of control group was found to reduce as compared to normal group which was significantly increased after treating animals with curcumin. This indicates that the depressed animals show less exposure and locomotion due to fear and anxiety where the animal prefers to stay immobile and steady by isolating into one place giving least number of ambulation counts [14, 15].

OBX induced depression is reported to be associated with over activity of Hypothalamus-Pituitary-Adrenal (HPA) axis as evident by over expression of CRF neurons. The activation of HPA axis leads to the release of CRH and ACTH to compensate stress which ultimately causes the excessive release of cortisol. In the present study, the serum cortisol level in OBX control group was found to be increased and was significantly attenuated in standard and test groups which is in-line with the previous findings [40, 41]. As previously reported the anti-anxiety effects as well as serum cortisol lowering by curcumin SMEDDS can be corroborated to its HPA axis amelioration effects. Curcumin have already proposed as anti-depressant by *Kulkarni et al.* [14] and *Chang et al.* [13], probably by inhibiting monoamine oxidase enzyme and modulating the release of serotonin and dopamine. In the present study, the behavioral parameters showed improve in memory, cognition and locomotion in curcumin (SMEDDS) groups, also the serum cortisol level was decreased as compared to test groups (pure curcumin) which are supporting to the findings made by *Kulkarni et al.* [14]. On the basis, data in hand and with support from literature therefore, it may be proposed that curcumin SMEDDS increased the brain permeability and biological activity as compared to the pure curcumin. Findings in the present study support the contention of various authors indicating the efficacy of nanoformulation over conventional drug delivery system for improved oral absorption and poor bioavailability of curcumin.

## 5 Conclusion

Conventional dose of curcumin SMEDDS significantly increased the locomotion in open field test and actophotometer, Improved memory and cognition in passive avoidance test as well as reduced serum cortisol level. These effects were considerably better than pure curcumin. Thus, it can be concluded that the nanoformulation of curcumin in the form of SMEDDS might be responsible for improved brain permeability and thereby enhancing its biological activity.

## Declarations

### Author contribution statement

Manoj Aswar, Mangesh Bhalekar: Conceived and designed the experiments; Analyzed and interpreted the data; Wrote the paper.

Akshata Trimukhe: Performed the experiments.

Urmila Aswar: Contributed reagents, materials, analysis tools or data.

### Funding statement

This research did not receive any specific grant from funding agencies in the public, commercial, or not-for-profit sectors.

### Competing interest statement

The authors declare no conflict of interest.

### Additional information

No additional information is available for this paper.
